# Percutaneous Mechanical Aspiration in Infective Endocarditis: Applications, Technical Considerations, and Future Directions

**DOI:** 10.1016/j.jscai.2023.101269

**Published:** 2024-02-20

**Authors:** Abdallah El Sabbagh, Evin Yucel, David Zlotnick, John M. Moriarty, Stephanie Younes, Nadira Hamid, Yasir Akhtar, Larry M. Baddour, Patrick O’Gara, Christoph Starck, Sripal Bangalore, Sahil A. Parikh, Kenneth Rosenfield, Sanjum S. Sethi

**Affiliations:** a Department of Cardiovascular Medicine, Mayo Clinic, Jacksonville, Florida; b Division of Cardiovascular Disease, Corrigan Minehan Heart Center, Massachusetts General Hospital, Harvard Medical School, Boston, Massachusetts; c Division of Cardiovascular Medicine, University of Buffalo, Gates Vascular Institute, Buffalo General Medical Center, Buffalo, New York; d Department of Interventional Radiology, University of California, Los Angeles, California; e Division of Cardiovascular Diseases, ProMedica Toledo Hospital, Toledo, Ohio; f Minneapolis Heart Institute, Abbott Northwestern Hospital, Minneapolis, Minnesota; g Department of Cardiovascular Diseases, Tennessee Heart Clinic, Knoxville, Tennessee; h Division of Public Health, Infectious Diseases and Occupational Medicine, Departments of Medicine and Cardiovascular Medicine, Mayo Clinic, Rochester, Minnesota; i Division of Cardiovascular Disease, Department of Internal Medicine, Brigham and Women’s Hospital, Boston, Massachusetts; j Department of Cardiothoracic and Vascular Surgery, German Heart Center of Charité, Berlin, Germany; k Division of Cardiovascular Medicine, New York University Grossman School of Medicine, New York, New York; l Division of Cardiology, Department of Medicine, Columbia University Irving Medical Center, New York

**Keywords:** cardiovascular implantable electronic device, endocarditis, injection drug use, outcomes, percutaneous mechanical aspiration, tricuspid valve

## Abstract

In recent years, there has been a shift in the epidemiology of patients with infective endocarditis (IE). This has been characterized by an alarming increase in IE in patients who inject drugs, cardiac implantable electronic device–related IE, and those with comorbid conditions and high surgical risk. This unmet need has mandated a reevaluation of complex management strategies in these patients and introduction of unconventional approaches in treatment. Percutaneous mechanical aspiration has emerged as both a diagnostic and therapeutic option in selected patients with IE. In this review, the authors discuss the gaps in care of IE, rationale, device armamentarium, procedural, and technical considerations and applications of percutaneous mechanical aspiration in IE.

## Central Illustration

**Figure:**
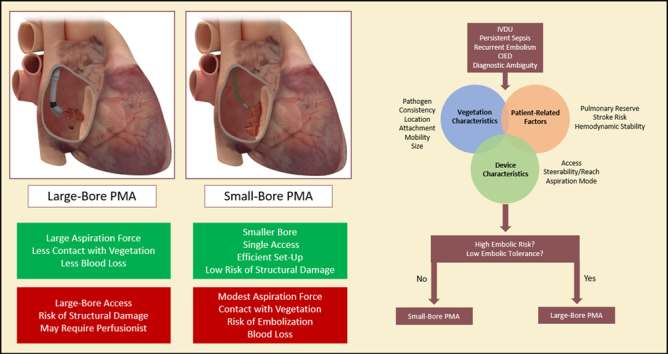

## Introduction

Infective endocarditis (IE) remains an epidemiologic burden with a rising incidence, affecting 3 to 10 per 100,000 patients per year, and comprising 40,000 to 50,000 new cases annually in the United States.^1^, ^2^, ^3^ Historically, the conventional standard of care for IE consisted of antimicrobials and surgery.^4^ The recent and rising opioid epidemic has led to a sharp increase in the incidence of injection drug use–related IE (IDU-IE),^5^ estimated to associate with a 400% increase in hospitalization rates from 2005 to 2016 with most cases involving right-sided IE.^6^ The rise of IDU-IE poses a challenge in conventional treatment paradigms because of the recurrent substance use and reinfection after surgical valve replacement, along with a financial and health care burden associated with the rise in hospitalizations.^7^^,^^8^ Hence, alternatives to conventional approach have been sought. The expansion of percutaneous thrombectomy techniques for the treatment of pulmonary embolism has led to interest in the off-label use of percutaneous mechanical aspiration (PMA) in IE that may not be amenable to conventional management strategies.^9^^,^^10^ The purpose of PMA is cardiac imaging–guided catheter-based extraction/debulking of vegetations with the goal of enhancing the efficacy of antimicrobial therapy with clearance of bloodstream infections in refractory septicemia, lowering the risk of septic embolization, reducing valve destruction and its hemodynamic consequences, and potentially lessening hospital length of stay. By delaying or avoiding surgical valve replacement, the risk of prosthesis reinfection may also be decreased.^6^ PMA has evolved as an option in the contemporary team–based approach in management of IE, and its use in right-sided IE received a class IIb recommendation in the 2023 European Society of Cardiology guidelines for management of IE.^11^ PMA in IE has also been cited as an option in a recent Scientific Statement from the American Heart Association^12^ and the European Heart Rhythm Association consensus document^13^ for management of right-sided IE. Several devices and techniques for PMA are currently available and predominantly used in right-sided IE. In this review, we discuss the gaps in diagnosis and treatment of IE and highlight the role of PMA. We describe the tools and technical approaches in PMA of IE and discuss outcomes and risks, as well as future directions for the field.

## Gaps in diagnosis and management of IE

There has been a shift in the spectrum of patients affected by IE. Patients with IE with previous cardiac surgery, congenital heart disease, immunocompromised conditions, organ transplantation, hemodialysis with vascular access devices, injection drug use (IDU) and cancer have increased in prevalence.^14^ Moreover, there has been a rapid increase in implanted cardiac devices^15^ and transcatheter intracardiac therapies.^16^^,^^17^ In parallel, an increased incidence of device-related endocarditis has occurred.^15^, ^16^, ^17^ These changes in epidemiology of IE have posed diagnostic and therapeutic challenges. A diagnosis of IE relies on clinical presentation, positive blood cultures, and imaging, particularly, echocardiography.^18^ A confirmed diagnosis can be achieved by histologic examination of the vegetation, which is not available in each patient.^19^ Establishing an accurate diagnosis and initiating therapy are fundamental because delayed diagnosis is associated with adverse clinical outcomes.^20^ Therefore, methods to establish a definitive diagnosis for those who are labeled as “possible IE” are essential for early and effective treatment. In addition, among cases of polymicrobial IE in persons who inject drugs (PWID), isolates obtained from blood cultures may not reflect the polymicrobial nature of IE.^21^ Other challenges in IE management include the risk of adverse drug reactions due to antibiotic therapy^22^ and the financial burden of prolonged hospital stays. As such, there has been a drive to find ways to shorten the duration of intravenous antibiotics and hospital stay, while maintaining or enhancing the response to treatment.^23^

The other arm of conventional therapy has been surgery in selected patients performed for 3 main indications: persistent infection despite antimicrobial therapy, structural damage, and recurrent embolic risk.^24^ Surgery is deferred in many patients felt to be at very high or prohibitive risk for complications including those with significant comorbidities and PWID who are at risk of infecting their prosthetic valve in case of recurrent drug use.^21^ In fact, a recent study showed that only 9.1% of PWID underwent tricuspid valve (TV) surgery and experienced an associated high rate of major adverse cardiovascular events.^25^ Moreover, the timing of surgical intervention has been a matter of ongoing debate. The benefit of delaying surgery is to allow better sterilization and stabilization, which lessens the risk of reinfection of prosthetic valves.^26^ At the same time, delaying surgery runs the risk of disease progression with recurrent embolization, structural damage, heart failure, abscess formation, and death, counteracting the benefits of prompt surgery and can be a nidus for reinfection as in the case of embolization.^20^ PMA may have a potential role in these gaps and aid with diagnostic sampling of vegetations in addition to enhancing response to antibiotics, lowering the risk of embolization, and serving as definitive treatment in high surgical risk patients or a bridge to surgery or transcatheter intervention.

## The rationale behind PMA in IE

The pathophysiology of IE starts with bacteria entering the bloodstream and adhering to damaged cardiac endothelium or foreign objects. Following adhesion, there is bacterial proliferation along with an immune inflammatory response with thrombus formation giving rise to vegetation.^27^ With organisms being concentrated in the center of the vegetation, surrounded by thrombus along with formation of a biofilm, host immunity and antimicrobial penetration are attenuated.^28^ Vegetation growth and extension then ensues, causing damage to cardiac structure. The rationale behind PMA is to reduce the size of the vegetation disrupt immune evasion and improve antibiotic efficacy, lowering the risk of embolic events, and potentially to prevent structural damage to the valve.

## Applications of PMA: reasons for intervention

**Figure 1:**
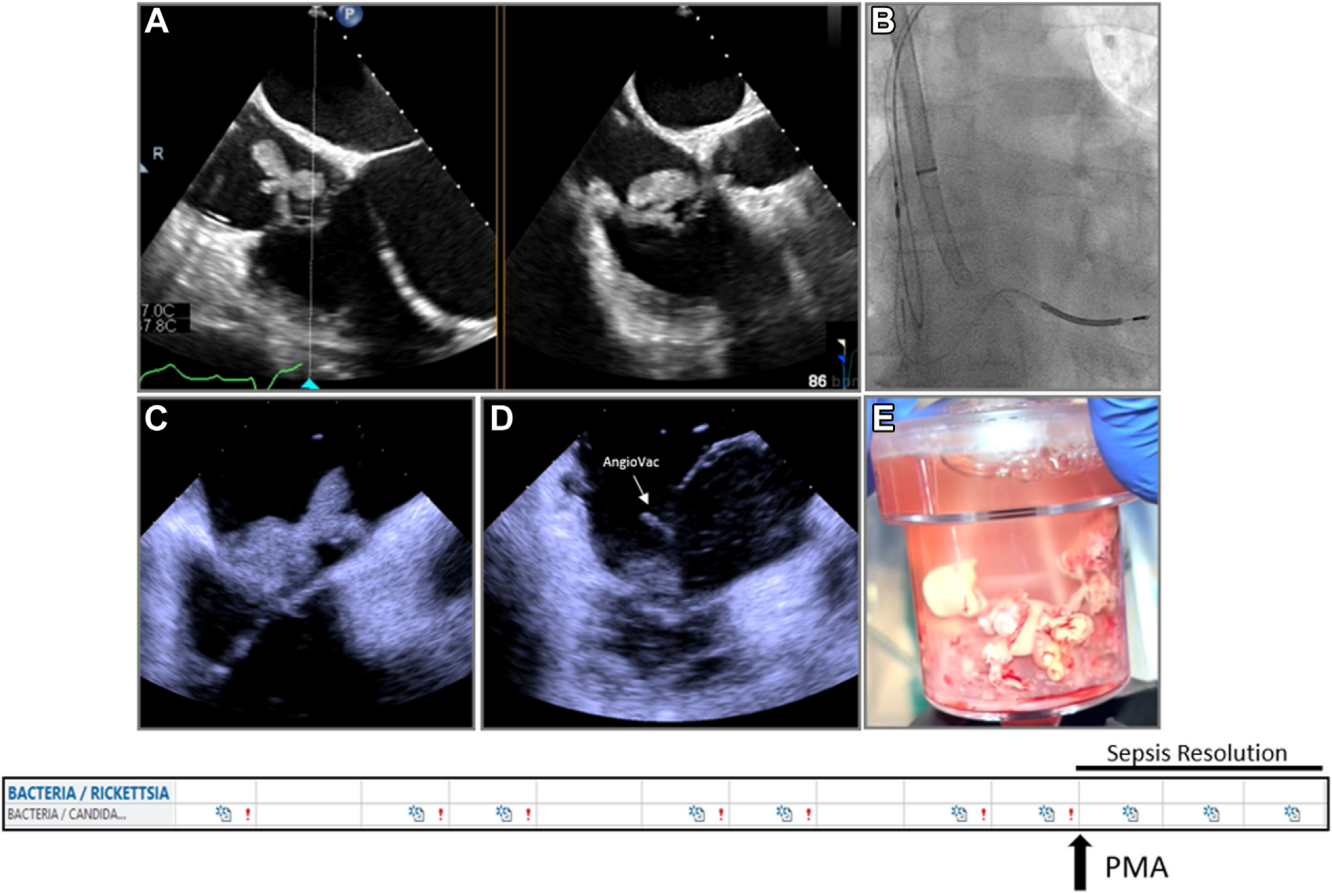
**Percutaneous mechanical aspiration in persistent sepsis despite antimicrobials.** Patient with large tricuspid valve vegetation (**A**) with persistent sepsis despite antimicrobials underwent large-bore aspiration using AngioVac under fluoroscopic (**B**) and intracardiac echocardiography (**C,D**) with effective debulking (**E**) and resolution of the sepsis.

**Figure 2:**
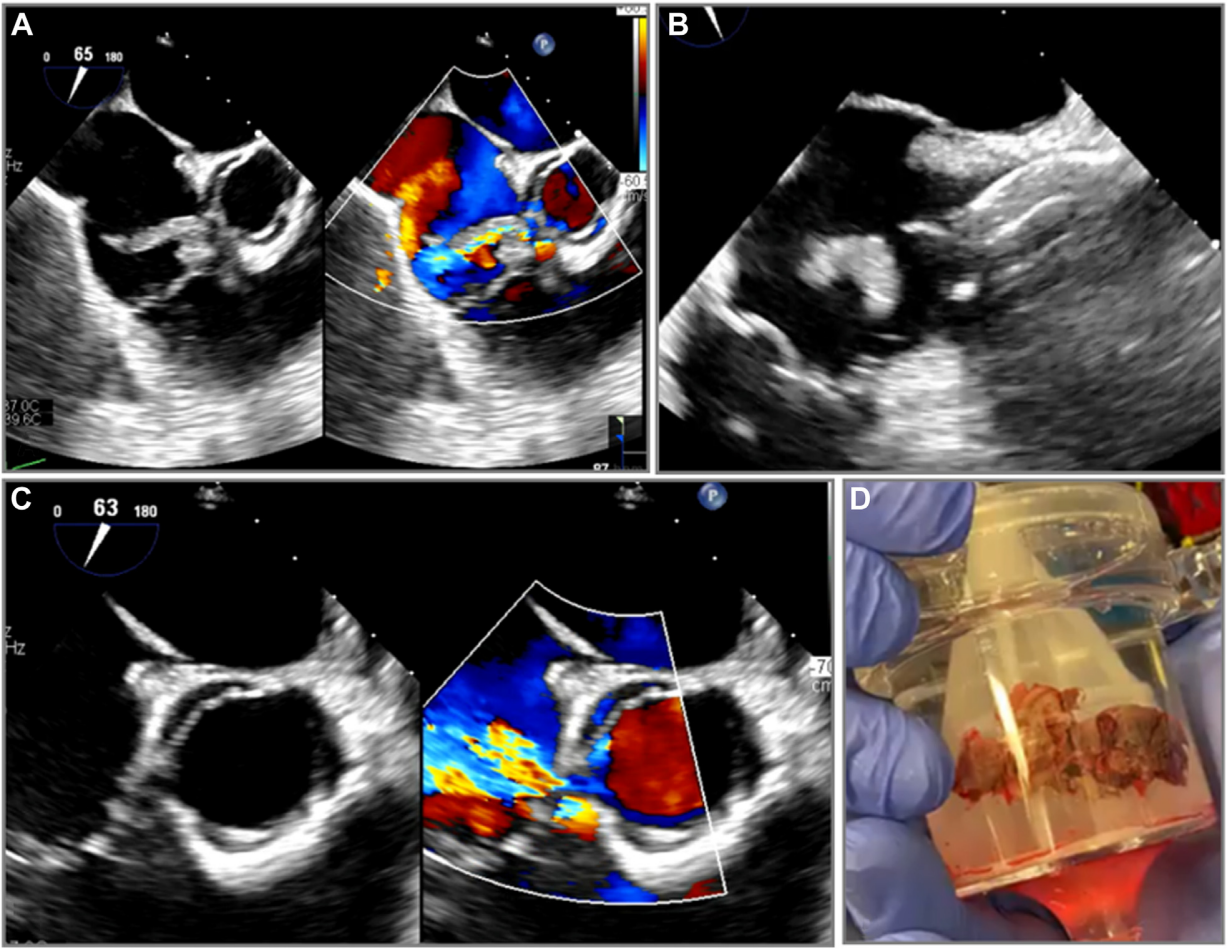
**Percutaneous mechanical aspiration in perforated tricuspid valve.** Patient with a large tricuspid valve vegetation from injection drug-use related infective endocarditis and pre-existing perforation in the base of the tricuspid valve leaflet (**A**) underwent transesophageal-guided large bore aspiration using AngioVac (**B**). Post aspiration, there was worsening tricuspid valve regurgitation from uncovering of the leaflet perforation (**C**). Specimen was collected and sent to pathology (**D**).

**Figure 3:**
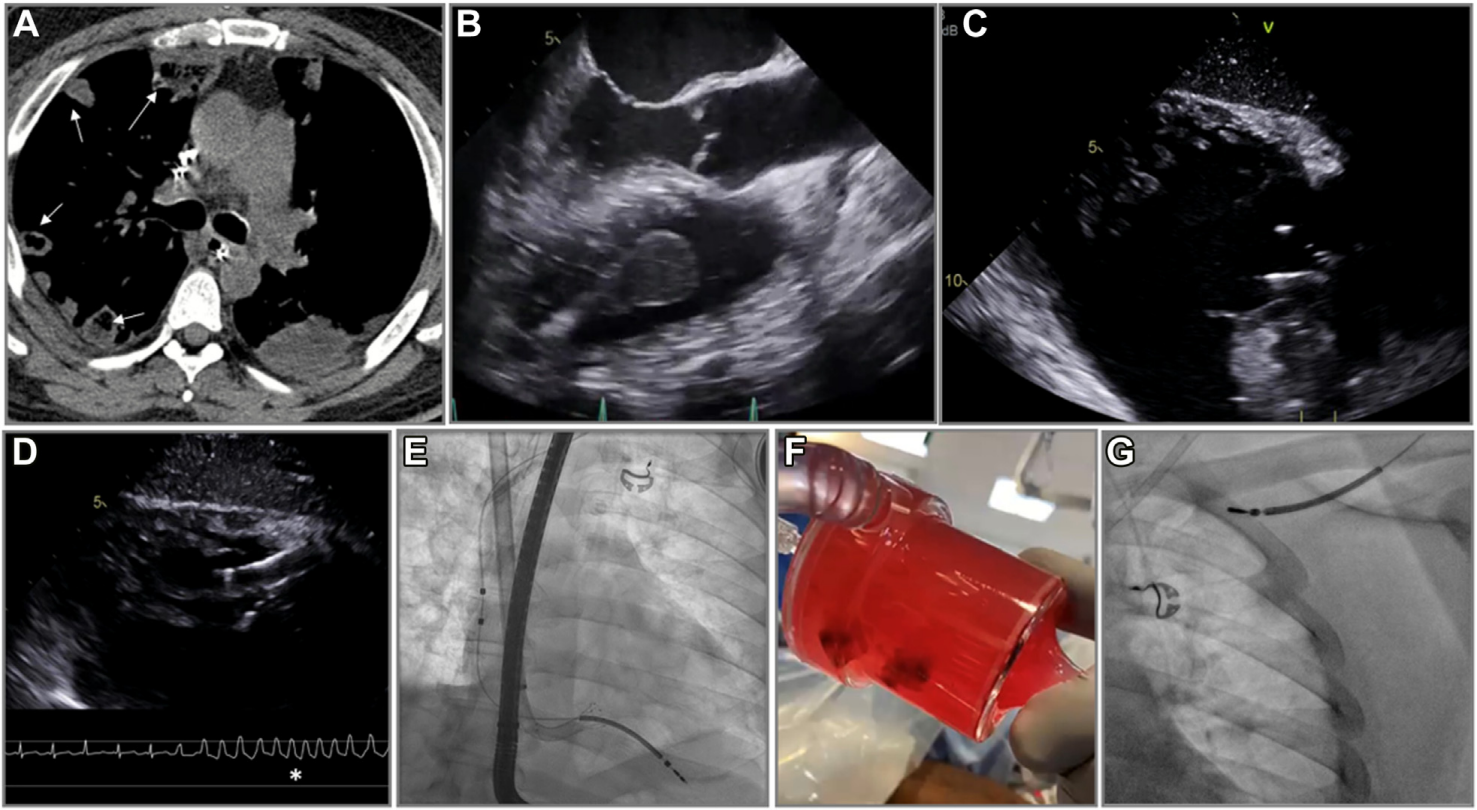
**Percutaneous mechanical aspiration in cardiac implantable electronic device related infective endocarditis.** Patient presented with recurrent pulmonary septic emboli despite antmicrobials (**A**, white arrows). There was a large vegetation on the right ventricular lead (**B**). Patient underwent percutaneous mechanical aspiration using AngioVac under transesophageal (**C,D**) and fluoroscopic (**E**) guidance, with a run of ventricular tachycardia due to a suck down event that resolved with repositioning the catheter (**D**). The vegetation was successfully aspirated (**F**) and led to safe lead extraction (**G**).

**Figure 4:**
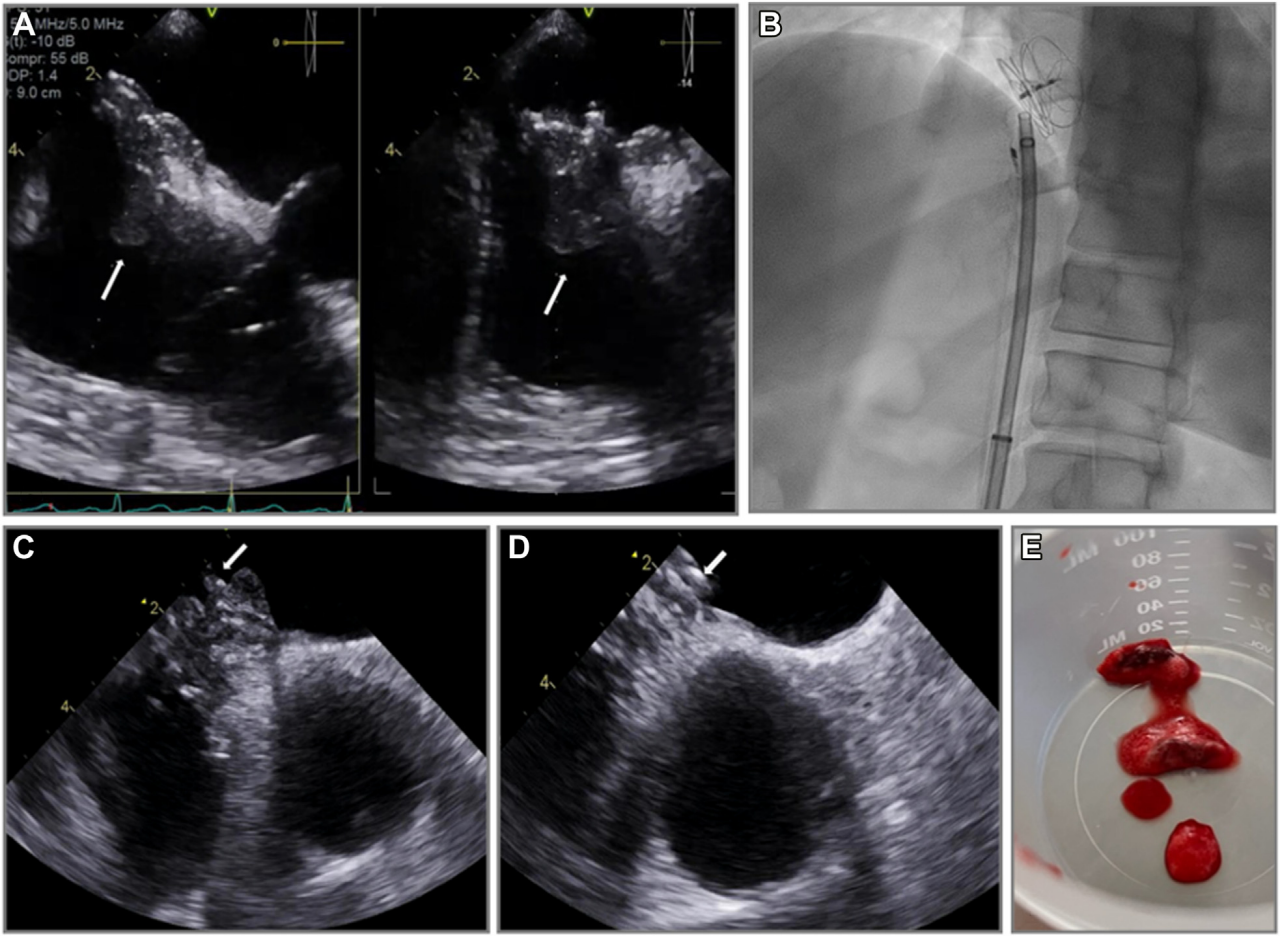
**Percutaneous mechanical aspiration for diagnostic sampling.** Patient with ambiguous echodensity on patent foramen ovale closure device (**A**, white arrows) underwent small-bore percutaneous mechanical aspiration using fluoroscopy (**B**) and intracardiac echocardiography guidance (**C,D**) with retrieved specimens consistent with thrombus and not infective endocarditis (**E**).

Currently, PMA is considered as a potential option in high-risk cases in which conventional therapy is not deemed feasible or appropriate. Currently, indications and timing of intervention are often based on expert opinion in each site and are areas of further investigation. The most common reasons to proceed with PMA are summarized in Table 1. Surgical results are often diminished owing to continued IDU and reinfection.^7^ Therefore, there is increased use of PMA in PWID-related IE.^6^^,^^9^^,^^10^^,^^29^^,^^30^ Several case series have shown feasibility of PMA with antibiotics in this patient subset, as destination therapy (Figure 1) or as a bridge to surgical valve intervention if structural damage has ensued^9^^,^^10^^,^^29^, ^30^, ^31^ (Figure 2). Delaying surgical intervention allows time for treatment of the core underlying problem, which is the addiction, through rehabilitation, thus reducing the chance for reinfection of a newly implanted prosthesis.^6^ PMA has been also been used in patients who have persistent sepsis or recurrent embolization who are not surgical candidates^9^^,^^10^^,^^29^^,^^31^^,^^32^ and in those with cardiac impantable electronic device (CIED) and lead vegetations that are ≥20.0 or ≥10.0 mm with a patent foramen ovale (PFO).^33^ Septic pulmonary emboli as a complication of both CIED infection and its removal can increase the combined risk of morbidity and mortality and may also present as a nidus of reinfection for newly implanted devices.^33^^,^^34^ In this setting, PMA allows for safer lead extraction, lowering the risk of septic embolization (Figure 3). PMA can also play a role in tissue sampling in cases of ambiguous diagnostic data (Figure 4). Several centers have established multidisciplinary endocarditis teams to help guide management of patient with IE (Table 2).^6^ The members of an endocarditis team may vary based on the interests, expertise, and resources present in a given institution and/or environment.

**Table 1:** Applications of percutaneous mechanical aspiration in infective endocarditis.

Intravenous drug use
Persistent sepsis despite adequate antibiotics
Recurrent septic embole despite adequate antibiotics
Cardiac implantable electronic device infection/facilitating lead extraction
Diagnostic ambiguity

**Table 2:** Multidisciplinary endocarditis team.

Cardiology
Infectious diseases
Interventional cardiology
Cardiac surgery
Interventional radiology
Vascular surgery
Neurology
Addiction medicine
Nutrition specialists
Physical therapy
Pulmonology

## Approach to patient and device selection

Case and corresponding device selection rely on the knowledge of device features, reason(s) for the procedure, vegetation characteristics, and patient-related factors (Central Illustration).

**Central Illustration:**
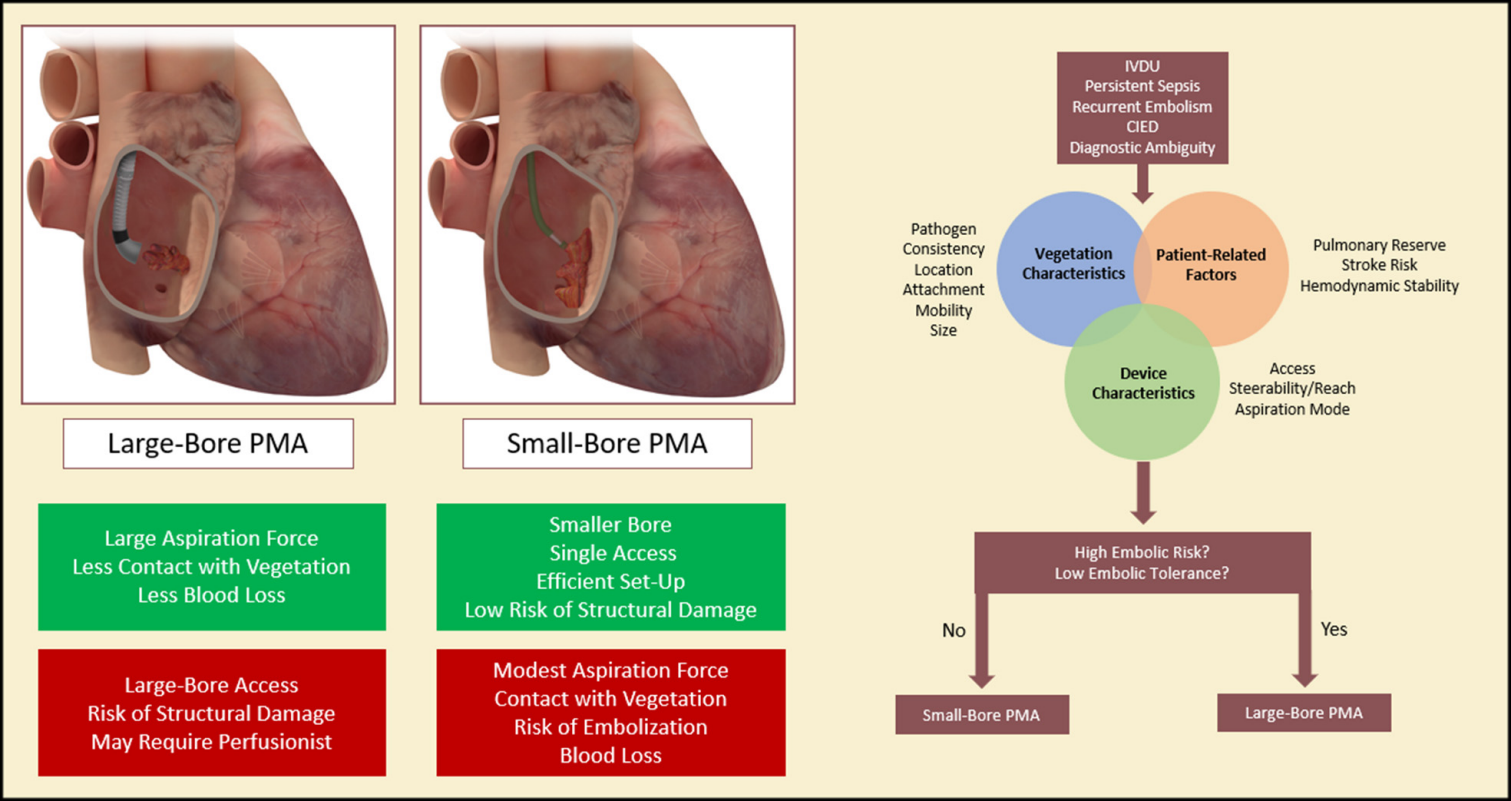
Algorithm and device selection for PMA of IE. (Left) Advantages (green) and disadvantages (red) of large-bore and small-bore PMA of IE. (Right) Suggested approach to device selection in PMA for IE. CIED, cardiac impantable electronic device; IE, infection endocarditis; IVDU, intravenous drug use; PMA, percutaneous mechanical aspiration.

### PMA: device features

In general, PMA devices are divided into 2 categories: large-bore and small-bore aspiration devices (Central Illustration). Large-bore aspiration devices use large size sheath catheters and generate substantial aspirating forces to pull blood through the catheter tip and a filter to collect the material. This substantial pulling force is useful for large vegetations and may lower the risk of distal embolization during the procedure. On the other hand, small-bore aspiration devices generate modest aspiration forces and require contact with the vegetation, followed by negative pressure aspiration or fragmentation of the material. Each of these type of devices have their advantages and disadvantages, and operators have to be familiar with the characteristics of these devices (Table 3).

**Table 3:** PMA device characteristics.

Access and reach
What is the size of the access sheath and PMA catheter?
What is the length and reach of the PMA catheter?
Steerability and flexibility
Does the intended aspiration catheter have the steerability and flexibility to be coaxial with the vegetation?
Aspiration mode
What is the mechanism of aspiration of the PMA catheter?
What is the device aspiration power?
Does aspiration require contact with the vegetation?
Does the device have a blood return mechanism?

PMA, percutaneous mechanical aspiration.

### Large-bore aspiration devices

The available large-bore aspiration devices for PMA are the AngioVac (AngioDynamics), currently in its third-generation iteration, the AlphaVac (AngioDynamics), and the FlowTriever (Inari Medical).

**Figure 5:**
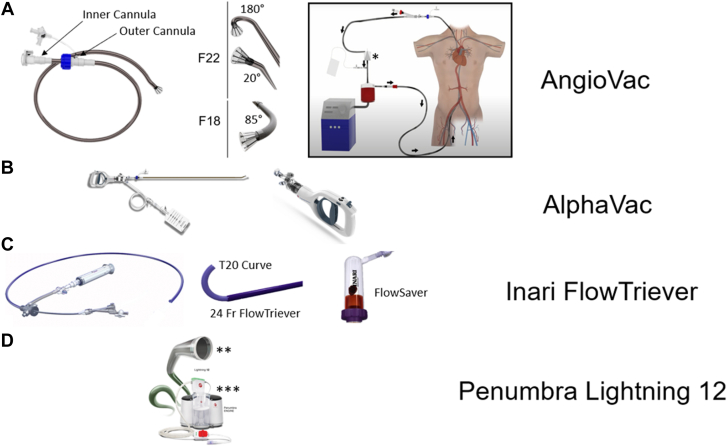
**Device armamentarium in percutaneous mechanical aspiration in infective endocarditis.** AngioVac system (**A**) consists of outer and inner cannulas that are connected to a perfusion circuit and return cannula. AlphaVac (**B**) uses a handheld mechanism for aspiration, while Inari FlowTriever (**C**) uses negative pressure generated by a large syringe and has a blood return mechanism using FlowSaver. Penumbra Lightning 12 (**D**) uses engine-generated aspiration forces.

The AngioVac device is composed of an outer cannula, through which an inner cannula with a funnel at its tip is introduced and extruded in a telescoping fashion (Figure 5A). There are 2 sizes of the device: F22 and F18. The inner cannula for the F22 is a 22F inside a 25F outer cannula system and comes in 2 shapes: 20° or 180°, signifying the proximal bend of the catheter that forms after extrusion from the outer cannula (Table 4). The 180° cannula has more degrees of freedom, and the angle and orientation can be changed by telescoping the inner cannula through the outer cannula. On the other hand, the F18 AngioVac system has an 18F inner cannula with an 85° bend on the distal tip, inside a 22F outer cannula. The inner cannula length for the F22 AngioVac system is 77.0 cm, while the F18 has a 105.0-cm length, hence more reach. Moreover, the inner cannula for all AngioVac systems have a side port that allows introduction of wires and equipment. Aspiration is initiated when the extracorporeal membrane oxygenation (ECMO) circuit is initiated, and blood is drawn via the inner cannula. This blood is then passed through a filter that traps solid material and air bubbles, followed by returning filtered blood via the return sheath. The vast majority of cases are done with no oxygenator or blood warming in the circuit; however, it is possible to connect an oxygenator in the case of concomitant respiratory failure. Advantages of this device is that the aspiration force is continuous, strong, and adjustable based on the circuit ramp speed, minimizing the need for contact between the catheter and the vegetation for successful aspiration, and this may lower the risk of embolization during the procedure. Moreover, given the blood return, blood loss is minimal using this device. For these reasons, this device remains the most commonly used in cases of PMA in IE. Disadvantages include the large-bore size of the catheters with vascular and structural complications associated with that, along with cost and the logistical challenges in the set up. This system often requires either a dedicated perfusion team or other team members who are trained for placing patients on bypass circuits. The availability of such persons may limit the use of this device in certain situations.

**Table 4:** Percutaneous mechanical aspiration devices used in infective endocarditis.

Device	Access	Cannula size	Length (cm)	Steerability	Aspiration mechanism	Aspiration power	Blood return	Advantages	Disadvantages
Large bore									
AngioVac F22	26F	25F outer; 22F inner	77.0	++	V-V or V-A ECMO	+++	Yes	Strong continuous aspiration forces; lower embolic risk; blood return	Vascular injury risk; structural damage risk; learning curve; cost and logistical challenges in setup; perfusionist availability
AngioVac F18	22F	18F	105.0	++	V-V or V-A ECMO	+++	Yes		
AlphaVac F22	26F	25F outer; 22F inner	77.0	++	Manual aspiration (handle)	++	No	Efficient setup; single access; strong pulling force	Vascular injury risk; intermittent aspiration; contact with vegetation (risk of embolization); no blood return mechanism
AlphaVac F18	22F	18F	105.0	++	Manual aspiration (handle)	++	No		
Inari FlowTriever	22-26F	20F or 24F outer and 20F T20 curve	95.0 (20F and 24F) and 113.0 (T20 curve)	+	Manual aspiration (syringe)	+++	Yes	Efficient setup; single access; strong pulling force; blood return	Vascular injury risk; structural damage risk; flow power not continuous
Small bore									
Penumbra Lightning 12	12F steerable sheath	12F	115.0	+++	Engine mediated	+	No	Efficient setup; less risk of vascular injury; less risk of structural damage; steerability; maneuverability	Modest aspiration forces; need for contact with vegetation (risk of embolization); no blood return mechanism

The AlphaVac is another large-bore aspiration system with similar design to the 18F and 22 F AngioVac systems (Figure 5B). The difference to the AngioVac system is that the end of the inner cannula is connected to a handle that when pulled in a quick manner, creates a high negative aspirating force if the cannula funnel is fully sealed by the vegetation. The amount of blood aspirated with each plunge can be controlled (10 and 30 mL) but is not returned. The advantage of this system is the efficient setup without the need for a perfusionist and strong pulling force when the cannula funnel is fully sealed by the vegetation. The main disadvantage is the embolic risk because of the intermittent nature of the aspiration and need for contact between the large cannula and the vegetation, as well as the blood loss since the device has no blood return mechanism.

Another current large-bore aspiration device is the FlowTriever (Figure 5C). The FlowTriever system works by connecting negatively charged 60-mL aspiration syringe to the back end of the aspiration catheter, with a luer lock that can be partially or fully open to generate different aspiration forces. The syringe containing the aspirated blood and vegetation can then be connected and emptied into the FlowSaver, which is a reservoir that can filter the blood from the retrieved specimen and reinfuse it back into the patient to minimize blood loss. The large-bore FlowTriever comes in different sizes (20F and 24F), with a flexible sheath. Through the 24F FlowTriever, a T20 curve catheter can be inserted. The T20 curve catheter has a 260° bend at the proximal end, which provides telescoping degrees of freedom depending on how much of the T20 catheter exits the outer 24F FlowTriever. The advantages of the system include the easy setup, with no need for a perfusion team, along with the minimal blood loss. The disadvantage of using this system is that the aspiration syringe generates strong aspiration forces at the beginning, but those forces diminish as the syringe is filled with blood; therefore, flow power is not continuous. Other disadvantages include the need for catheter manipulation and the use of shaped wires to deliver the catheter, particularly in right ventricular (RV) vegetations. The misalignment can pose a risk for suction induced injury to the cardiac chambers and vessels.

### Small-bore aspiration devices

Small-bore aspiration requires catheter contact with the vegetation, aspirating it either as a whole or fragmented. There is 1 commonly used small-bore aspiration device: Penumbra Lightning 12 catheter (PL12).

The PL12 is a 12F catheter with 115.0 cm in length and a soft atraumatic tip, connected to an engine that generates the negative pressure for intermittent aspiration (Figure 5D). The PL12 is inserted through a 12F steerable sheath. Aspiration is initiated by turning on the engine. There are pressure sensors built into the engine to provide real-time audio and visual feedback on the flow monitoring. If the vegetation is in the catheter, interrupting aspiration, the sensor changes color and the audio queue stops, signifying the need to remove the catheter to flush it. Advantages of this system is the low profile, steerability, and maneuverability, particularly with the steerable sheath, as well as the ease of setup and continuous suction at vacuum. Disadvantages include the modest aspiration forces during aspiration and the need to contact the vegetation and lack of blood return mechanism. Device features are summarized in Table 4.

### Pathogen and anatomic features

Bacterial vegetations are the predominant targets of PMA, particularly those due to *Staphylococcus aureus*, which is the most common (80%) pathogen in right-sided IE in PWID. *S aureus* is also a leading cause of CIED-related IE.^24^ Right-sided IE due to fungi (mostly *Candida* species) may recur, and some may disseminate systemically owing to a contact with a PMA catheter, complicating the clinic presentation and treatment goals. Therefore, the microbiology of IE may influence procedural considerations, and more data are needed to assess its risks on PMA.

The anatomic characteristics of the vegetation are also important to assess for case planning. Anatomic evaluation includes assessing for vegetation consistency, location, attachment, mobility, and size (CLAMS). Vegetations in an acute IE presentation have a soft consistency and are more amenable to percutaneous debulking, whereas chronic calcific vegetations, especially sterile vegetations, are less likely to effectively be removed. In right-sided IE, vegetations in locations near the inferior vena cava, superior vena cava, right atrium, atrial side of the TV, middle and apical RV, and ventricular side of the pulmonic valve are amenable to PMA, whereas the ventricular side of the TV and pulmonic side of the pulmonic valve are unfavorable locations owing to inability to position the aspiration catheter effectively or safely. Vegetation attachment, mobility, and size are important to assess the risk of embolization. A large vegetation, defined as ≥20.0 mm with a stalk that is highly mobile likely carries a higher embolic risk compared with a smaller sessile vegetation with a broad base. Large, highly mobile vegetations that form acutely with a narrow stalk are more favorable for PMA than chronic, diffuse, and sessile vegetations.

### Embolic risk and tolerance

The next step in case planning is assessment of the embolic risk and tolerance to embolic events during the procedure based on patient-related factors, vegetation characteristics, and device features. Large vegetations that are highly mobile may carry higher embolic risk during the procedure. Tolerance to embolic events is also important to assess and depends on patient characteristics and comorbidities. In right-sided IE, the embolic tolerance in the pulmonary vasculature is usually acceptable, except in patients who have a poor baseline lung reserve, such as those who present with multiple septic emboli. Moreover, patients with right-sided vegetations and a PFO are at risk of stroke with PMA, and use of cerebral embolic protection device can be considered. In such high-risk patients with high procedural embolic risk and low tolerance to embolic events, large-bore aspiration catheters would generate large suction forces without making full contact with the vegetation, theoretically lowering the risk of distal embolism (Central Illustration).

## PMA: role of imaging for procedural planning and guidance

**Figure 6:**
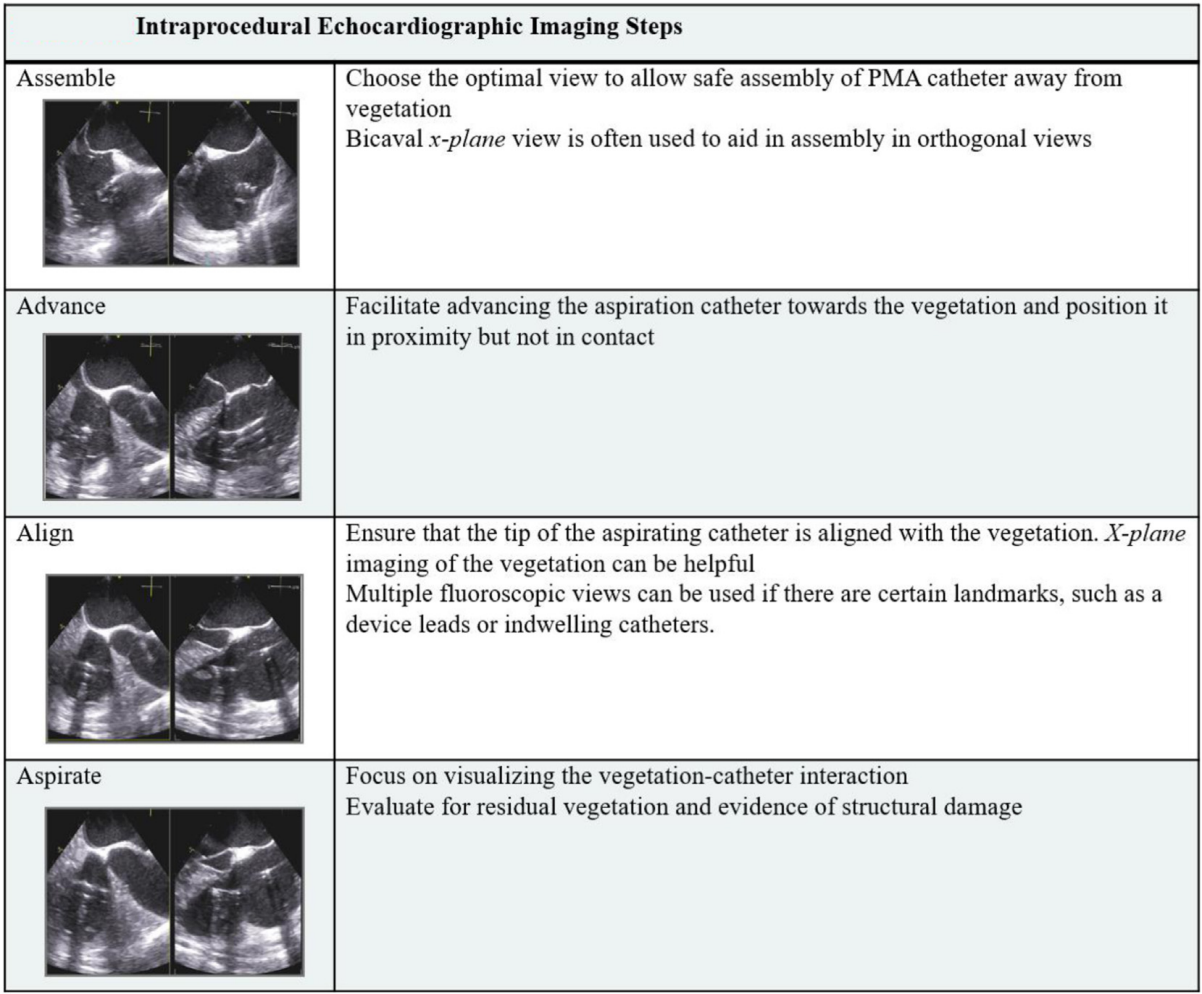
Intraprocedural echocardiographic imaging steps.

Imaging plays an essential role in procedural planning and guidance of PMA in IE. Prior to the procedure, a transesophageal echocardiogram (TEE) is recommended to assess the characteristics of the vegetation, which in turn would help evaluate the procedural risks (Table 5).^35^^,^^36^ Baseline imaging should also identify the presence of valvular regurgitant lesions and its mechanism because PMA may worsen regurgitation, possibly by uncovering areas of perforation. As with most contemporary cardiac procedures, a common language between the imaging and procedural specialists is essential to ensure a synchronized and systematic approach. Intraprocedural imaging can aid in assembling devices inside the heart chambers, avoid suction injury, and help avoid embolization of vegetation by visualizing device proximity to the vegetation. Intracardiac echocardiography (ICE) can also be used, depending on the operator preference and the risk of interaction of the ICE catheter with the vegetation. The imaging sequence to ensure synchrony with procedural steps is summarized in Figure 6.

**Table 5:** Preprocedural imaging assessment of vegetations.

Vegetation size
Measurement of maximal length of vegetation in its longest axis by 2D echocardiography in different views
Vegetation mobility
Absent mobility: fixed with no independent motion
Low mobility: fixed base with mobile free edges
Moderate mobility: pedunculated but remains within the same chamber during cardiac cycle
Severe mobility: prolapses across the coaptation plane of the leaflets during the cardiac cycle
Vegetation consistency
Echogenicity
Echolucency
Calcification
For example, a heterogeneous echogenic or highly echolucent vegetations suggest an acute process, whereas calcification suggests a chronic vegetation
Valvular regurgitant lesions
Mechanism
Quantification of severity

## Procedural step-by-step approach

### Right-sided IE

#### Large-bore aspiration

##### AngioVac system

Large-bore aspiration using AngioVac system requires a team consisting of an endovascular/surgical specialist, anesthesiologist, perfusionist, and cardiac imager. From a sedation standpoint, these procedures are done predominantly with general anesthesia, but conscious sedation in select cases can be utilized. Factors that favor general anesthesia are as follows: need for TEE guidance; patient comfort, particularly when the internal jugular vein is used as a large-bore access site for the cannulas; and respiratory status of the patient at baseline, for example, if they are at risk of decompensation with procedural pulmonary septic embolism or pulmonary edema that may occur after returning the blood from the circuit toward the end of the procedures (as in patients with low ejection fraction).

**Figure 7:**
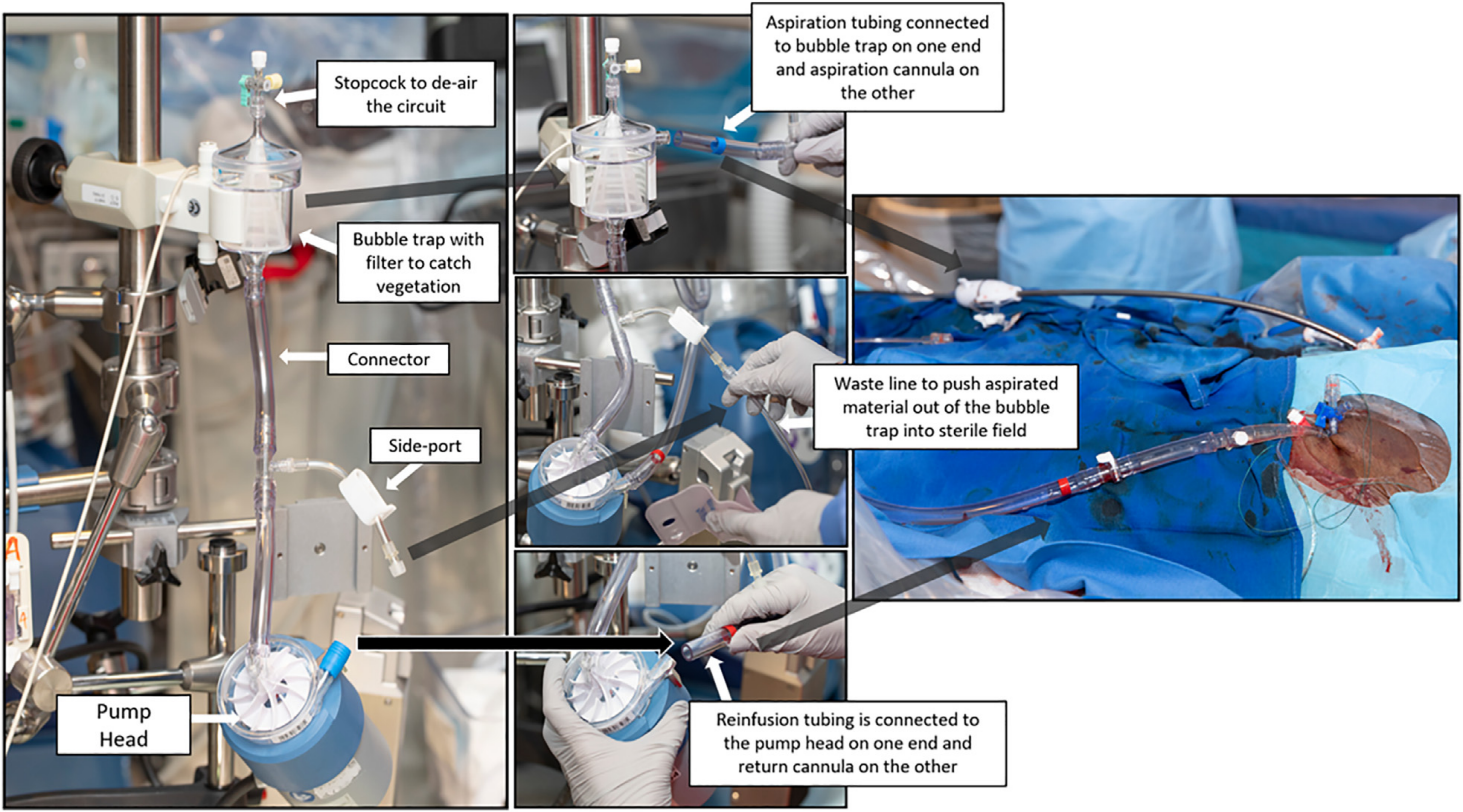
**Establishing the AngioVac circuit.** PMA, percutaneous mechanical aspiration.
7 The outer aspirating cannula is inserted into the sheath over its dilator and stiff wire. The dilator is then removed, and the inner aspirating cannula is introduced through the outer cannula in a wet-to-wet fashion.
8 As a rule of thumb, it is prudent to extrude the inner cannula funnel through the outer cannula before circuit flow is initiated to avoid infolding of the inner cannula funnel inside the outer cannula. Extrusion of the inner cannular funnel is performed under imaging and fluoroscopic guidance and has to be completed distant to the vegetation to avoid inadvertent interaction. Funnel extrusion is performed in the right atrium for both TV and RV vegetations.
9 Once the funnel is extruded, circuit flow is initiated at low speed and the inner cannula funnel is advanced slowly toward the vegetation. If the vegetation is in the RV, funnel extrusion is performed in the right atrium, and flow is initiated at low speed, which allows floating of the cannulas through the TV. Manipulation of the inner and outer cannulas are often required to provide adequate reach and angle to align with the vegetation and is performed under imaging guidance. In case of RV vegetations, delivering the cannula across the TV can be challenging owing to cannula bias toward the base of TV and depending on the caval-tricuspid angle. This can be tackled by carefully floating a balloon wedge catheter into the pulmonary artery to deliver a stiff wire, over which the AngioVac cannula can be delivered into the right ventricle.
10 Once the inner cannula is in proximity to the vegetation and aligned in 2 views on echocardiography (or x-plane TEE across the vegetation can be performed), circuit flow is ramped up, while the funnel is advanced gently toward the vegetation. The circuit flow is then increased gradually until the vegetation is aspirated. As the circuit speed is ramped up, the operator has to watch for suck-down events, particularly in the RV apex where the catheter is close to the RV wall. Suck-down events are detected by a tactile thump, arrhythmia, or slowing of circuit flow, and in such circumstances, flow speeds are turned down, and the catheter is carefully repositioned.
11 Circuit speed is then decreased, and the site of the vegetation is imaged in multiple views to check for residual vegetation. The definition of successful debulking has been accepted by several studies as reducing the vegetation volume by 70% or more without having procedural complications such as death, vascular injury, sustained arrhythmias, valvular damage, or embolic events.^10^^,^^33^ In the case of CIED explantation with lead extraction, certain operators keep the cannula in the lower right atrium with a constant circuit flow as the lead is being extracted to aspirate any residual vegetation that can be retained or dislodged in the venous vasculature. Adjunct snaring of residual vegetation is not usually recommended in the index procedure and can be staged if the sepsis and/or embolic events continue despite debulking. The latter is typically performed through a separate access point to introduce the snare while suction is on. Finally, techniques that involve intentionally dislodging the vegetation with a catheter while suction is on with the hopes of aspirating the dislodged vegetation are not recommended given the risk of distal embolization.
12 After the vegetation is debulked, the aspiration cannula is removed and pointed upward outside the body to prevent air from entering the system. Blood is then slowly returned (using gravity), and the return cannula is disconnected. In patients with low ejection fraction, care has to be taken with the rapidity of blood reinfusion or administration of extra bolus of saline to return the blood because it can lead to pulmonary edema.
13 Then, the sheaths are removed, and hemostasis is achieved. Heparin is reversed if needed.
14 The vegetation is removed from the filter and measured (to the best possible if fragmented) and sent to pathology and microbiology for analysis.

Because large-bore aspiration relies on volume, hypotension may occur in patients who are being overdiuresed or overdialyzed and may require fluid infusions at the time of the procedure. The procedure starts by access planning. Selection of the access sites to establish the venovenous circuit depends on the location of the vegetation. The most commonly used venous access site configurations for aspiration and return cannulas are femoral-femoral or internal jugular vein-femoral. In general, vegetations are approached from the side opposite to its location to allow alignment of the aspiration cannula with the vegetation. For example, vegetations near the superior vena cava are approached from the femoral site via the inferior vena cava and vegetations near the inferior vena cava are approached via the right internal jugular vein and superior vena cava. Vegetations on the posterior TV leaflet are best approached from the internal jugular vein approach, whereas those on the anterior and septal leaflets may be approached from a femoral or internal jugular venous access. Vegetations in the right ventricle are best approached from a right internal jugular vein access, although femoral approach can be used for more basal-mid ventricular locations. After deciding access sites, next step is obtaining access and establishing the circuit through the following step-by-step approach:

1 Ultrasound-guided venous puncture.
2 Unfractionated heparin is given to achieve an activated clotting time of >250 seconds.
3 Preclosure of access site: Suture-based vascular occlusion devices has been used in several series, although there is a hypothetical risk of access-related infection given the IE.^29^ Another option would be to proceed with a figure of eight or purse string suture in a preclosure fashion, which would serve not only to establish hemostasis after procedure, but also to help and cinch the large-bore sheaths in place.
4 Over a stiff wire, the venous punctures are dilated progressively, followed by placement of a 26F sheath for the F22 AngioVac cannula and 22F sheath for the F18 Fr AngioVac cannula. A 16F, 17F, or 18F reinfusion cannula is inserted into the second venous access site to serve as the blood return.
5 The outer and inner cannulas are assembled outside the body in a telescoping fashion. To facilitate telescoping and maneuvering the inner cannula through the outer cannula, the outer cannula is flushed with a fat emulsion preparation such as Propofol or RotaGlide, and the inner cannula is exercised through the lubricated outer cannular prior to insertion into the access sheath. This would minimize the friction between the outer and inner cannulas.
6 The return cannula is connected to the centifugal pump console through tubing in a wet-to-wet fashion to minimize trapped air. The inner aspirating cannula is connected to the other limb of the circuit (Figure 7).

**Figure 8:**
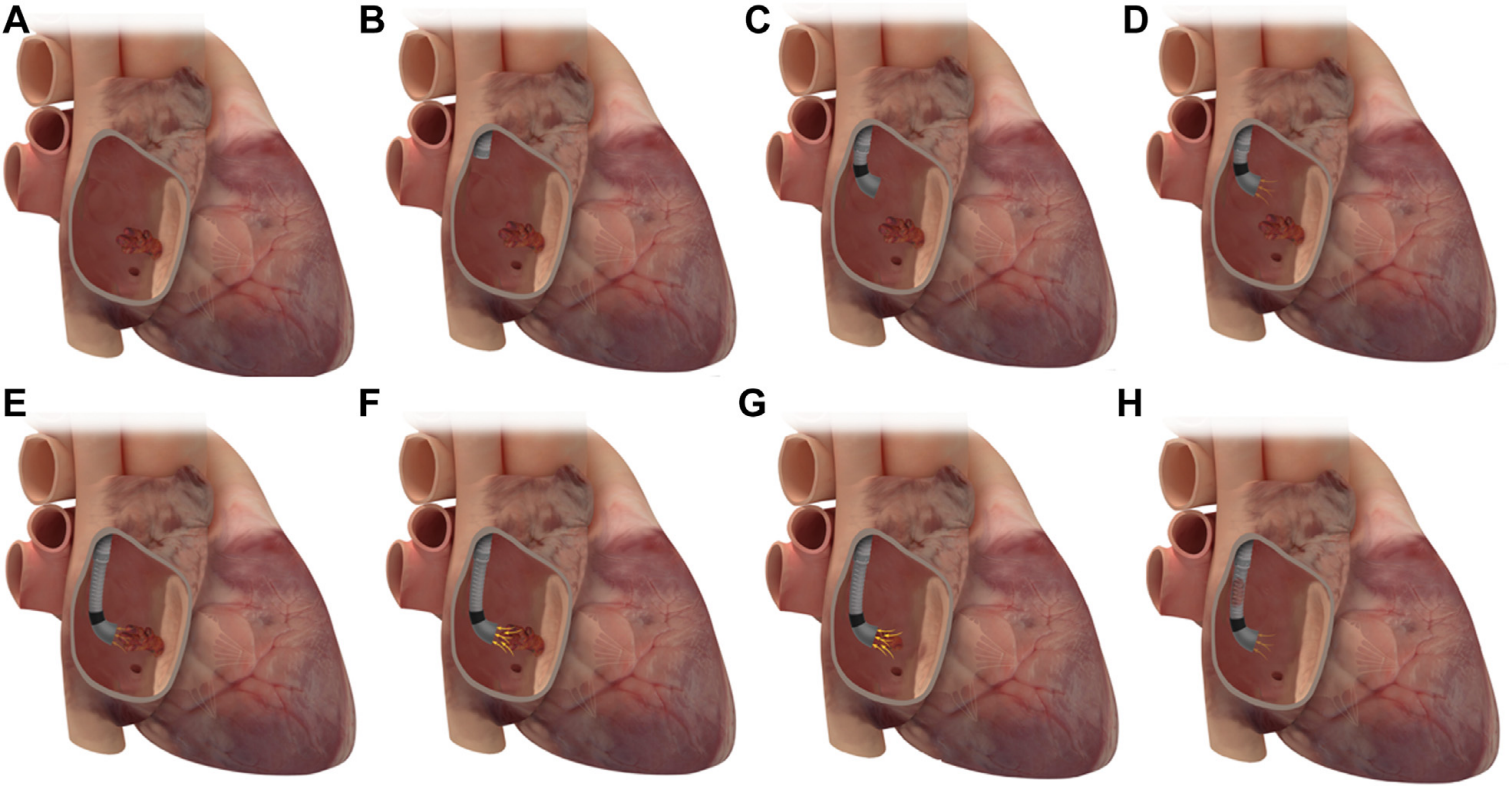
**Large-borePMA of a right-sided IE using AngioVac device.** Large-bore PMA of right-sided IE (**A**) using AngioVac device. The outer cannula is first introduced (**B**), followed by the inner cannular (**C**). Circuit flow is then initiated at low speed (**D**), and with continuous aspiration on, the AngioVac cannula is advanced and aligned in proximity to the vegetation (**E**), and the flow speed is then ramped up (**F**), generating high aspiration forces, successfully aspirating the vegetation (**G,H**). IE, infectious endocarditis; PMA, percutaneous mechanical aspiration.

Step-by-step right-sided PMA using AngioVac is summarized in Figure 8 and Supplemental Video 1.

##### AlphaVac system

The procedural steps of large-bore aspiration using the AlphaVac system are similar to those described earlier for the AngioVac system with a few exceptions. Given the mechanism of aspiration using AlphaVac as described earlier, high aspiration forces are generated mostly when the vegetation seals the inner cannula funnel. Therefore, it is recommended to approach the vegetation while aspiration is being generated by frequent manual pulls until contact is made with the vegetation. The amount of blood aspirated and lost with each pull can be controlled on the handle and can vary between 10 and 30 mL. In general, 10-mL pulls are used to approach a vegetation, whereas 30-mL pulls are used to generate effective forces to aspirate after contact is made.

##### FlowTriever

In the case of large-bore debulking using the 24F FlowRetriever with the T20 inner catheter, there are some differences in the approach compared with that of AngioVac. Access is from a single site. Aligning the 24F FlowTriever catheter with the vegetation can be challenging and often requires keeping a wire, carrying some risk of dislodgement of the vegetation. Extruding the T20 curve catheter can help to further align the catheter with the vegetation. It is preferred to attempt and align the catheter with the vegetation as best as possible to allow effective debulking while minimizing the risk of catheter suction injuries to the valve and chamber walls because the aspiration force generated by the FlowTriever is strong. Once the catheter is aligned with the vegetation, certain operators recommend half-turn of the syringe leur-lock with short and partial aspirations to minimize suction-related injury. Once the specimen is retrieved, the blood can be filtered through the FlowSaver and reinfused into the patient.

#### Small-bore aspiration

**Figure 9:**
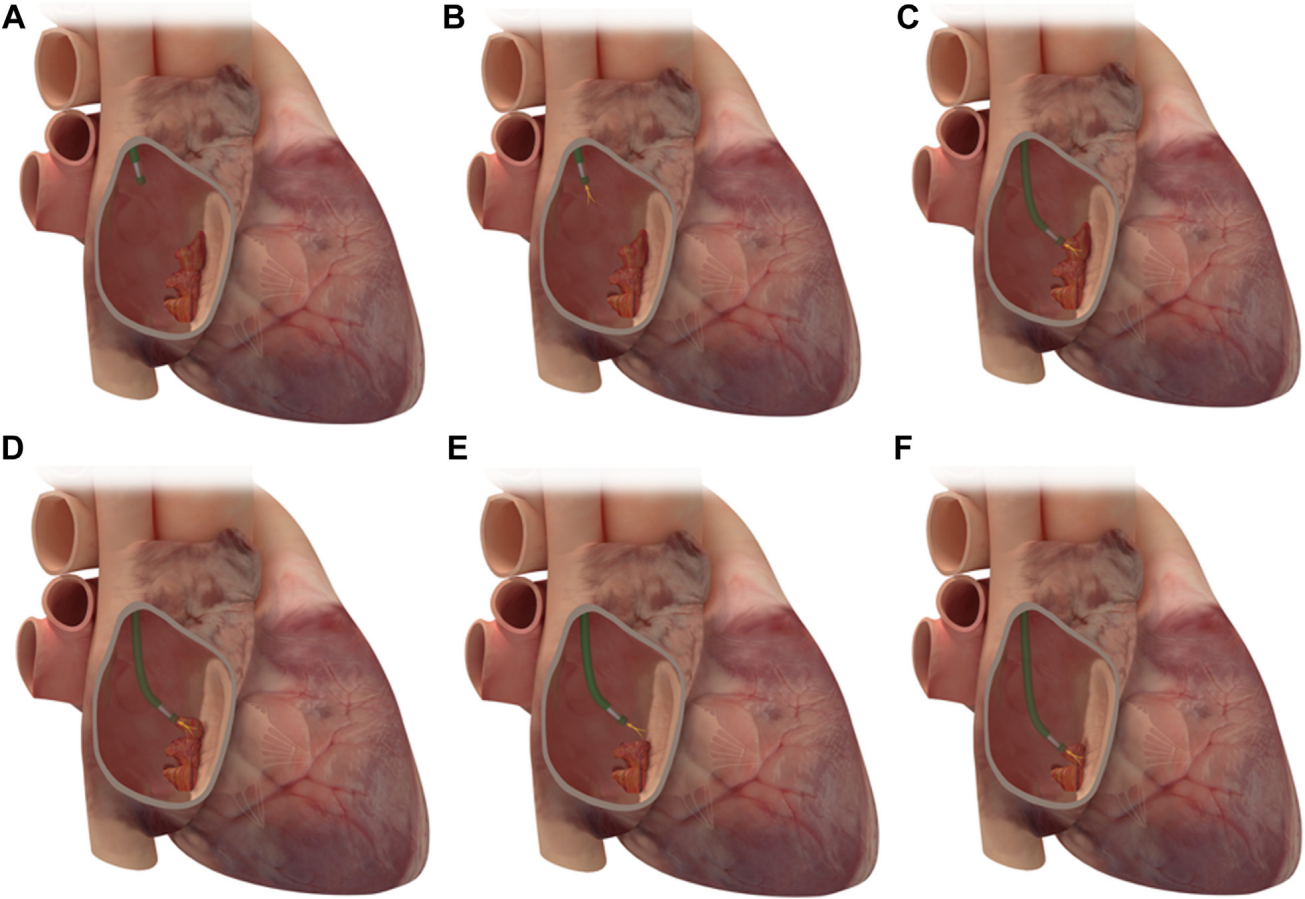
**Small-bore PMA using Penumbra Lightning 12**. (**A**) Once the device is introduced into the right atrium, aspiration is initiated (**B**), and the device is advanced until it engages and aspirates the vegetation (**C**). The device is then maneuvered to engage residual vegetation (**D–F**). PMA, percutaneous mechanical aspiration.

The procedural principles that drive the steps in small-bore aspiration are similar to those of large-bore aspiration despite the differences in the device characteristics. Owing to the more modest aspiration force, small-bore aspiration often requires contact with the vegetation to allow the catheter to latch and effectively aspirate. Access is obtained using the corresponding sheath for each device and site selection (internal jugular vs femoral vein) is similar to that discussed earlier. Once access is obtained, the aspirating catheter is extruded outside the sheath. For example, the PL12 catheter is extruded through a steerable 12F sheath. Once the aspirating catheter is extruded, it is placed in alignment with the vegetation by flexing and maneuvering the steerable sheath (Figure 9 and Supplemental Video 2). Aspiration is switched on by turning the aspirating engine in the Lightning system. Once aspiration is on, the catheter is advanced carefully under imaging guidance, and contact is made with the vegetation. This contact between the PL12 and vegetation is maintained to allow slow aspiration of the vegetation, either as a whole or through multiple passes depending on the characteristics of the vegetation. In devices that do not have a blood return mechanism, it is important to transfuse red blood cells for any identifiable anemia prior to the procedure and to consider stopping once ∼500 mL of blood is removed.

### Left-sided IE

**Figure 10:**
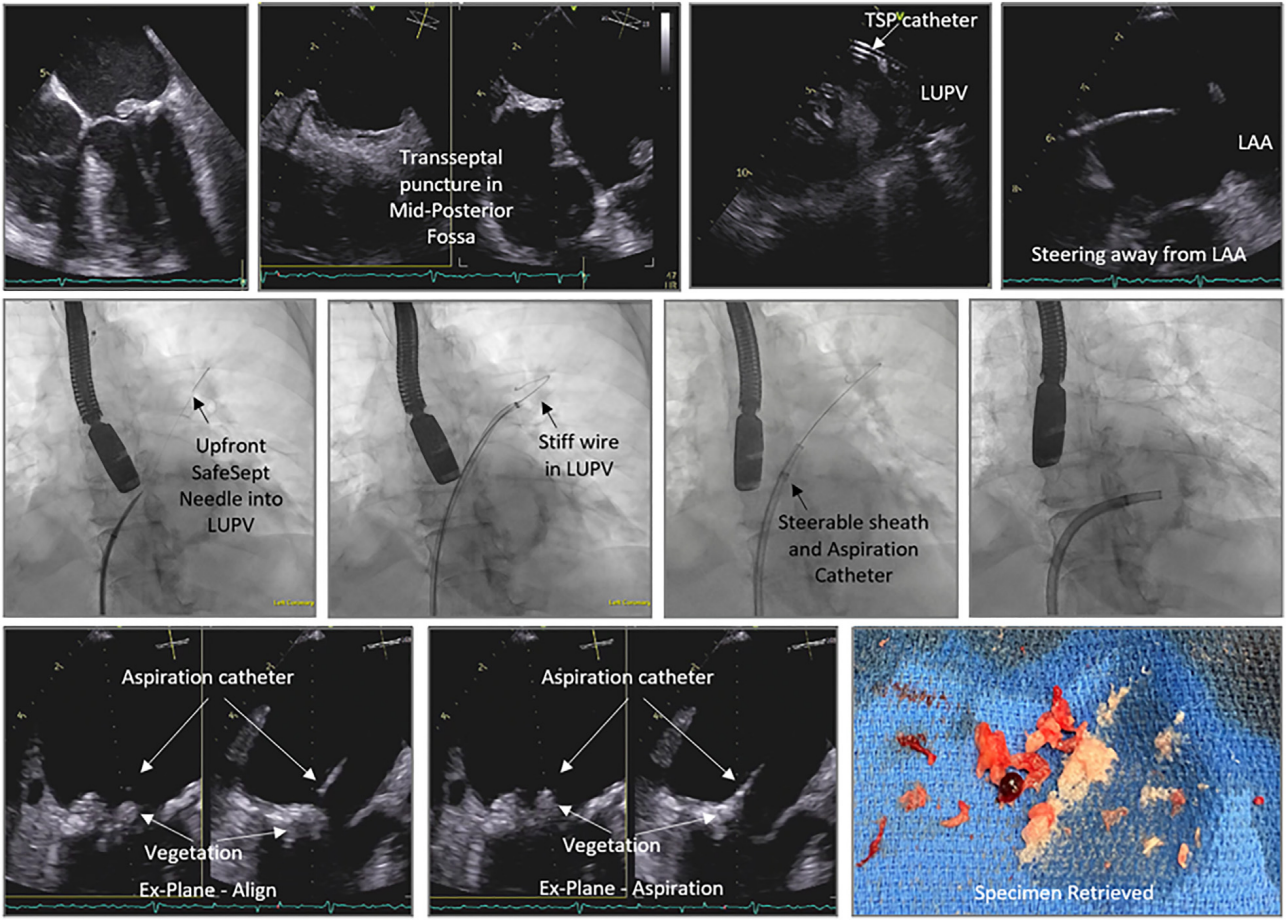
**Percutaneous mechanical aspiration of mitral valve infectious endocarditis via transseptal route.** LAA, left atrial appendage; LUPV, left upper pulmonary vein; TSP, transseptal puncture.

**Figure 11:**
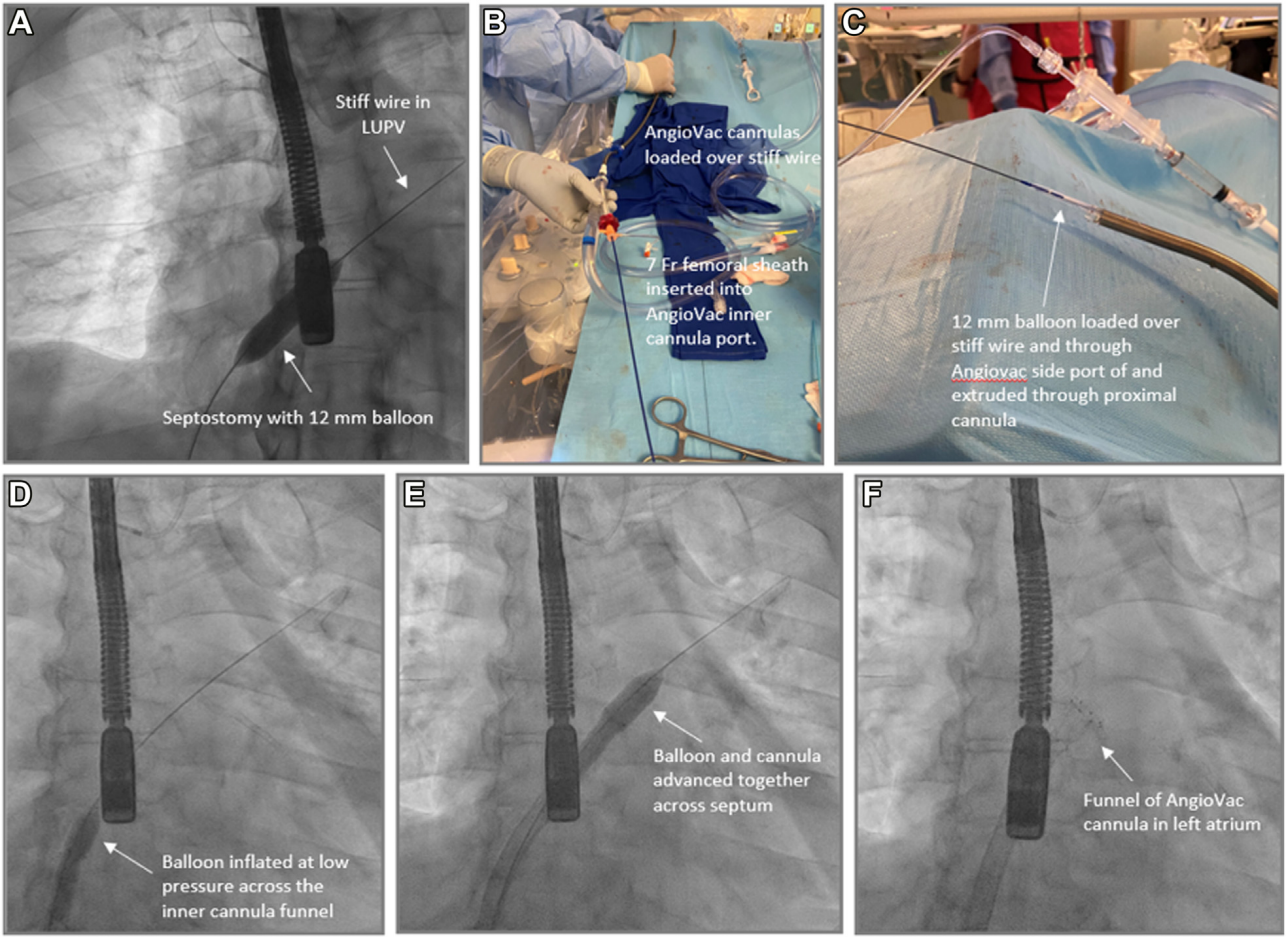
**Balloon-assisted tracking for transseptal delivery of AngioVac cannulas.** After performing a transseptal puncture and placement of a stiff wire in the left upper pulmonary vein (LUPV), a balloon septostomy is performed using 12 mm balloon (**A**). Following that, the AngioVac cannulas are loaded over the stiff wire and a 7 French sheath is inserted into the AngioVac inner cannula port (**B**), through which the 12 mm balloon is loaded and extruded through proximal cannula (**C**). The system is delivered into the right atrium and the balloon is inflated at low pressure (**D**) followed by advancement of the balloons and cannulas across the interatrial septum to remove the razor-effect from the cannulas (**E,F**).

PMA has predominantly been used in right-sided IE because the devices were designed for right-sided reach. However, PMA has been reported in left-sided IE.^37^ There is an area of unmet need in management of left-sided IE where surgery is not performed in ∼50% of patients, particularly in those with liver disease and stroke before surgery.^38^ In left-sided IE, vegetations on the atrial side of the mitral valve (MV) are amenable to PMA, whereas those on the ventricular side are unfavorable owing to limitations with reach. Aortic valve vegetations are also challenging and unfavorable, and retroaortic approach can be limited given the lack of reach and maneuverability of these devices in that location. Due to the risk of stroke, all patients with left-sided vegetations should have cerebral embolic protection. Transseptal route is the typical access for MV IE. For large-bore aspiration using AngioVac, cannula access is obtained in the right femoral vein and transeptally into the left atrium, and the return is usually to the left femoral vein. There is a risk of hypotension during circuit flow when blood is pulled from the left atrium and returned into a venous route. Hypotension can be avoided by using the femoral artery as the reinfusion access and maybe needed in cases of stroke where hypotension can be harmful. However, using the femoral artery as a reinfusion access comes at a risk of vascular injury and bleeding given the large-bore size sheath. Care must be undertaken with the transseptal puncture to avoid equipment dislodgement of the vegetation. We suggest using an upfront SafeSept wire to perform a transseptal puncture and direct the SafeSept wire into the left upper pulmonary vein (Figure 10). This creates a rail from the interatrial septum (IAS) into the left upper pulmonary vein and allows catheter and wire exchanges without interacting with the vegetation. Transseptal puncture is typically performed in a mid-posterior location on the fossa ovalis to have enough operating height over the MV vegetation. For the large-bore PMA devices, a septostomy is performed using a 12.0-mm balloon before advancing the aspiration catheters. For AngioVac, after the septostomy, the inner cannula is extruded through the outer cannula in the inferior vena cava over the stiff wire, and advanced across the IAS. Often, the funnel of the inner cannula catches on the IAS. This can be overcome by balloon-assisted tracking technique that takes the razor edge off the cannula funnel (Figure 11). For the PL12 system, the 12F steerable sheath is advanced across the IAS. Through that, the PL12 catheter is introduced into the left atrium. The catheter is then carefully oriented toward the MV vegetation using imaging guidance and making sure to steer past the left atrial appendage to avoid injury to the latter. The aspirating catheter is aligned with the vegetation in commissural TEE view for medial/lateral orientation and in left ventricular outflow tract view for anterior/posterior orientation. Finally, aspiration is performed using similar steps described earlier.

## Outcomes

Data on outcomes of right-sided PMA are limited to 2 registry studies and small case series (Table 6).^9^^,^^10^^,^^29^^,^^31^, ^32^, ^33^^,^^37^ Successful aspiration was defined as PMA of ≥70% of the vegetation^10^^,^^29^^,^^33^ The RAPID (The Registry of AngioVac Procedures in Detail) study was a multicenter registry of patients who underwent PMA of thrombus or vegetation using the second-generation AngioVac device.^10^ Of 234 patients, 59 patients had infected vegetations: 26 in the right atrium, 14 on the TV, 6 on CIED leads, and 1 in the RV. In this registry, 75% of cases experienced successful aspiration. Mortality was reported as 1.3% (3 patients).^10^ In another registry study, Starck et al^33^ evaluated the outcomes of PMA a using second-generation AngioVac in patients with CIED with large lead vegetations defined as ≥20.0 mm or 10.0-20.0 mm with a PFO. Most vegetations were on right atrial leads and less frequently in the ventricular leads. *Staphylococcus* species was the most common species in 55.4%. Procedural success was 90.4% and led to successful transvenous lead extraction in 99.2% of cases. In this registry, mortality rate at 30 days was 3%, which was low compared with published results on CIED explants in infections without the use of aspiration device.^33^^,^^39^ This generates the hypothesis of a survival benefit when performing a concomitant PMA procedure in such patients.

**Table 6:** Summary of registry data and case series for percutaneous mechanical aspiration in infective endocarditis.

Reference, year	Center	N	Endocarditis site	Vegetation size	Devices used	Follow-up	Procedural success (%)	Complications
Moriarty et al,^10^ 2021	Multicenter registry	59	Tricuspid valve; right atrium; right ventricle	-	AngioVac	In-hospital	75	Death 1.3%; distal embolization 3%; major bleeding 2.6%
Starck et al,^33^ 2020	Multicenter registry	101	CIED	30.7 ± 13.5 mm	AngioVac	30 d	94; lead extraction 99.2	Death 3%; distal embolization 1%
Zhang et al,^31^ 2023	New York University	29	Tricuspid valve	24 ± 7.6 mm	AngioVac	181 d	96.6	Death 6.9%; TVR 0%
Scantland et al,^29^ 2022	Indiana University School of Medicine	32	Tricuspid valve; indwelling device	3.2 cm^2^	AngioVac	60 d	90.6	TVR 16.5%; bleeding 3%
George et al,^9^ 2017	University of Kentucky	33	Tricuspid valve; tricuspid bioprosthetic valve	21.2 ± 7.0 mm	AngioVac	In-hospital	100	Death 9%; TVR 9%; major bleeding 3%
Akhtar et al,^32^ 2021	Tennova Heart Institute	25	Tricuspid valve	24.0 mm	Penumbra	1 mo	88	Death 12% (worsening septic shock); transfusion 28%
Akhtar et al,^37^ 2022	Tennova Heart Institute	3	Mitral valve	22.0 cm	Penumbra	2.5 mo	100	Stroke 1 patient; death 1 patient

CIED, cardiac impantable electronic device; ECMO, extracorporeal membrane oxygenation; TVR, tricuspid valve replacement.

In a recent case series, Zhang et al^31^ reported outcomes of PMA using AngioVac device in 29 patients with TV IE with average vegetation size of 24.6 ± 7.6 mm with staphyloccoci being the predominant organism. All patients in that series presented with septic emboli and had persistent sepsis despite antibiotics. Successful PMA was achieved in 96.6% of patients, evidenced by clearance of sepsis and mean vegetation size reduction of 71%, along with reduction of white blood cell count and mean body temperature after procedure. There were no intraprocedural deaths, but 2 patients (6.9%) died during the index hospitalization from complications related to necrotizing pneumonia. George et al^9^ reported that in 33 patients with IE, the average size of vegetation 2.12 ± 0.7 cm in longest dimension, who underwent PMA using a first-generation AngioVac device. The predominant pathogens were staphylococci, whereas 5 patients experienced polymicrobial infections and 5 candidemia. A residual vegetation was visible in 82.6% of patients, with an average size of 0.82 ± 0.5 cm. There were no intraprocedural deaths, but the rate of index hospitalization mortality was 9.1%.^9^ On the other hand, Scantland et al^29^ reported a series of 32 patients who underwent PMA using different generations of AngioVac. Successful PMA was achieved in 90.6%, with 84.4% of patients with histologic confirmation. Twenty-three patients presented with TV vegetations, 1 eustachian valve vegetation, 4 bioprosthetic TV vegetations, and 8 pacemaker leads and tunneled catheters. There were no deaths within 30 days.^29^ Finally, in a case series by Akhtar et al,^32^ 25 PWIDs underwent ICE-guided PMA using small-bore aspiration under conscious sedation. In this series, the average vegetation size was 2.4 × 1.5 cm. PMA reduced the vegetation by 77% ± 22% in all patients. All specimens showed bacterial colonies, predominantly gram-positive cocci, with no myocardial or valve tissue identified. Rate of death in this series was 8.3%.^32^ In another retrospective analysis by the same group, PWID who underwent PMA had similar 1-year all-cause mortality compared with that of patients who underwent valve surgery (24% vs 19%; *P* = .57).^30^

## Complications

### Vascular injury/blood loss

PMA can be associated with vascular injury and blood loss. In the RAPID registry, trauma at the cannula site occurred in 3.4% of cases, cardiac perforation in 0.4%, and arrhythmias in 1.3%. Major hemorrhage occurred in 2.6% of cases, with a hemoglobin change of −1.0 ± 1.5 g/dL per procedure and median hemoglobin decrease from 10.2 to 9.1 g/dL.^10^ In the registry of Starck et al,^33^ there was 1 patient who presented with right iliac vein perforation that was treated with covered stent. In the most recent case series by Zhang et al,^31^ there were no reported bleeding complications with the AngioVac device and hemoglobin dropped from 8.6 ± 0.21 g/L to 8.0 ± 0.18 g/dL (*P* < .01). In the case series by George et al,^9^ 1 (3%) patient developed cardiac tamponade and another (3%) experienced severe access site bleeding requiring transfusion (3%). Scantland et al^29^ reported 1 (3%) neck hematoma in their series. Finally, in the series by Akhtar et al,^32^ which reported the use of small-bore aspiration, there was no vascular injury, but given the lack of a blood return mechanism with the device used, there was an average blood loss of 0.5 ± 0.2 L, and 28% required blood transfusion after procedure. Blood loss with this device decreased temporally as the operator experience increased.

### Worsening valvular regurgitation

Worsening tricuspid regurgitation (TR) was demonstrated in several case series. George et al^9^ reported worsening TR in 43.5% of these patients, 3 of whom underwent TV replacement after clearance of blood cultures. Scantland et al^29^ reported 5 (15.6%) patients having subsequent TV surgery. Akhtar et al^32^ showed that a histologic review of retrieved specimen did not reveal valve tissue, raising the possibility that the reason for worsening TR is uncovering a perforation created by the IE, as opposed to direct damage related to the aspiration forces. Worsening TR in these interventions is usually tolerated for several weeks and, in case of IDU-IE, may allow referral to rehabilitation to treat the drug addiction before TV surgery. Larger studies are needed to verify the mechanism of worsening valvular regurgitation in patients undergoing PMA for IE.

### Distal embolism

In the RAPID registry, distal embolization occurred in 3% of cases, with 1 death related to inferior vena cava thrombus causing massive pulmonary embolism. Stroke occurred in 0.4% of cases.^10^ In the case series by George et al,^9^ 1 patient developed epidural abscess and spinal compression, leading to death. Finally, in a small series of MV endocarditis PMA using PL12, 1 patient died of stroke.^37^ Our suggested approach may help to lower the risk of distal embolization, with the principles of tailoring device selection with vegetation characteristics and size and approaching the target while aspiration is on and minimize contact with the vegetation during large-bore PMA. Larger studies are needed to evaluate the embolic risks of PMA. For right-sided lesions, pulmonary embolism extraction may be required to retrieve a procedure-related septic pulmonary embolus causing respiratory distress using the same or different aspiration device. In left-sided IE, stroke, coronary or peripheral artery interventional techniques may be required to retrieve embolized material if clinically indicated. Finally, embolic protection devices may potentially reduce risk of embolization with PMA in right-sided IE with PFO or left-sided IE.

### Worsening sepsis

Aspiration of a vegetation may lead to transient worsening sepsis, manifested by rigors and fever. In extreme cases, septic shock can occur and reported in the RAPID registry, with 1 patient death due to worsening septic shock,^10^ and in the registry by Starck et al,^33^ reporting 1 patient death due to intraoperative death related to refractory septic shock. In the case series by Akhtar et al^32^ using small-bore PMA, 3 patients died of worsening sepsis, and 1 patient required ECMO for shock and respiratory failure. This series also showed that among patients who initially presented with septic shock prior to the procedure, there was an associated increase in mortality. In event of hemodynamic compromise with any aspiration device, intropic support, and in extreme cases, V-A ECMO can be used. In case of AngioVac, V-A ECMO can be established by connecting the AngioVac return cannula to a Y connector, which is connected to the AngioVac venous return sheath, and a second ECMO circuit, which returns blood to a separate arterial access, establishing the V-A ECMO.^40^ It is also important to avoid upfront PMA prior to initiation of antimicrobial treatment and to continue it throughout the procedure. Further investigation of risks associated with worsening sepsis in patients who are candidates for PMA are needed.

## Future directions

PMA use in IE is on an accelerated trajectory. Large prospective registries and clinical trials are needed to inform indications and further define outcomes. Challenges exist in assessing longitudinal outcomes and clinical trial implementation in PWID owing to the substantial loss to follow-up, which is prevalent in this important group where PMA may be most beneficial. Clinical trials design in the CIED population undergoing PMA and device extraction compared to a standard-of-care cohort may provide procedural data and reliable follow-up owing to the need for device interrogation. Prospective registries can also be critical in the evaluation of the feasibility and safety of PMA in IE. They can also assess the impact of PMA on the natural history of IE compared with historical cohorts, including impact on source control, embolic risk, and structural valve integrity. PMA device–specific outcomes would also help further delineate the role of a specific device for a diversity of vegetation characteristics.

As the field continues to evolve, several potential applications may arise. For example, a recent clinical trial showed that early surgical intervention on large vegetations without embolic or structural damage was associated with improved survival.^41^ Moreover, a previous study showed that large (>10 mm) left-sided vegetations were associated with up to a 44% risk of embolic events.^42^ This raises the possibility of a potential role for early PMA of large vegetations as a preventative measure to avoid embolic complications and structural damage, shortening of antimicrobial duration and possibly improve surgical outcomes by enhanced tissue sterilization. This requires large studies to evaluate this potential benefit.

## Conclusion

The landscape of IE has changed and includes patients who are high or prohibitive surgical risk. PMA has emerged as a novel option in the diagnosis and treatment of valvular and CIED-related IE with a variety of devices available, each with their advantages and disadvantages. Further large-scale and prospective studies are needed to evaluate the indications for and outcomes with PMA in IE.

## Peer review statement

Associate Editor Sahil A. Parikh had no involvement in the peer review of this article and have no access to information regarding its peer review. Full responsibility for the editorial process for this article was delegated to Editor in Chief Alexandra J. Lansky.

## Declaration of competing interest

David Zlotnick is on the speaker’s bureau for Abiomed, Inari Medical. John Moriarty consults for AngioDynamics, Penumbra Medical, Innova Vascular, Pavmed, Pfizer, and Boston Scientific. Nadira Hamid consults for Abbott Structural, Anteris, AMX, 4C Medical Technologies, Alleviant Medical, Edwards Lifesciences, Philips, GE, Valcare Med, VDyne, and WL Gore. Yasir Akhtar received honoraria and is on speaker bureau for AngioDynamics and Penumbra. Kenneth Rosenfield consults for and/or is on the scientific advisory board member for Althea Medical, AngioDynamics, Boston Scientific, Contego, InspireMD, Magneto, Mayo Clinic, Neptune Medical, Philips, Summa Therapeutics, SurModics, Thrombolex, Terumo, and Truvic; holds equity in Accolade, Access Vascular, Aerami, Althea Medical, Contego, Cruzar Systems, Embolitech, Endospan, InspireMD, JanaCare, Magneto, Orchestra BioMed, PQ Bypass, Prosomnus, Shockwave Medical, Summa Therapeutics, Thrombolex, Truvic, and Valcare; and is a board member for National PERT Consortium. Christoph Starck received honoraria, consults for, and is on the advisory board of AngioDynamics, Abiomed, Atricure, Medtronic, Spectranetics, Biotronik, LivaNova (Sorin), and Cook Medical and received departmental or institutional research funding from Cook Medical and Hylomorph. Sripal Bangalore is on the advisory board for Abbott Vascular, Boston Scientific, Biotronik, Amgen, Pfizer, Merck, REATA, Inari Medical, and Truvic. Sahil Parikh receives institutional grants/research support from Abbott Vascular, Shockwave Medical, TriReme Medical, SurModics, Silk Road Medical, and the National Institutes of Health; has received consulting fees from Terumo and Abiomed; and has served on the advisory boards of Abbott, Medtronic, Boston Scientific, CSI, Janssen, and Philips. Sanjum Sethi reports honoraria from Janssen and Chiesi. Abdallah Sabbagh, Evin Yucel, Stephanie Younes, Larry Baddour, and Patrick O’Gara reported no financial interests.

## References

[1] Bor D.H., Woolhandler S., Nardin R., Brusch J., Himmelstein D.U. (2013). Infective endocarditis in the U.S., 1998-2009: a nationwide study. PLOS ONE.

[2] Federspiel J.J., Stearns S.C., Peppercorn A.F., Chu V.H., Fowler V.G. (2012). Increasing US rates of endocarditis with Staphylococcus aureus: 1999-2008. Arch Intern Med.

[3] Pant S., Patel N.J., Deshmukh A. (2015). Trends in infective endocarditis incidence, microbiology, and valve replacement in the United States from 2000 to 2011. J Am Coll Cardiol.

[4] Cahill T.J., Prendergast B.D. (2016). Infective endocarditis. Lancet.

[5] Ronan M.V., Herzig S.J. (2016). Hospitalizations related to opioid abuse/dependence and associated serious infections increased sharply, 2002-12. Health Aff (Millwood).

[6] Yucel E., Bearnot B., Paras M.L. (2022). Diagnosis and management of infective endocarditis in people who inject drugs: JACC state-of-the-art review. J Am Coll Cardiol.

[7] Lawrence C.H.D., Cheaveau J., Kavourides M., Chadwick D., McCarron B. (2021). Endocarditis and the impact of intravenous drug use: a cohort study. Infect Dis (Lond).

[8] Zubarevich A., Szczechowicz M., Osswald A. (2021). Surgical treatment of infective endocarditis in intravenous drug abusers. J Cardiothorac Surg.

[9] George B., Voelkel A., Kotter J., Leventhal A., Gurley J. (2017). A novel approach to percutaneous removal of large tricuspid valve vegetations using suction filtration and veno-venous bypass: a single center experience. Catheter Cardiovasc Interv.

[10] Moriarty J.M., Rueda V., Liao M. (2021). Endovascular removal of thrombus and right heart masses using the AngioVac System: results of 234 patients from the prospective, multicenter registry of AngioVac procedures in detail (RAPID). J Vasc Interv Radiol.

[11] Delgado V., Ajmone Marsan N., de Waha S. (2023). 2023 ESC guidelines for the management of endocarditis. Eur Heart J.

[12] Baddour L.M., Weimer M.B., Wurcel A.G. (2022). Management of infective endocarditis in people who inject drugs: a scientific statement from the American Heart Association. Circulation.

[13] Blomstrom-Lundqvist C., Traykov V., Erba P.A. (2020). European Heart Rhythm Association (EHRA) international consensus document on how to prevent, diagnose, and treat cardiac implantable electronic device infections-endorsed by the Heart Rhythm Society (HRS), the Asia Pacific Heart Rhythm Society (APHRS), the Latin American Heart Rhythm Society (LAHRS), International Society for Cardiovascular Infectious Diseases (ISCVID), and the European Society of Clinical Microbiology and Infectious Diseases (ESCMID) in collaboration with the European Association for Cardio-Thoracic Surgery (EACTS). Eur Heart J.

[14] Slipczuk L., Codolosa J.N., Davila C.D. (2013). Infective endocarditis epidemiology over five decades: a systematic review. PLOS ONE.

[15] Voigt A., Shalaby A., Saba S. (2006). Rising rates of cardiac rhythm management device infections in the United States: 1996 through 2003. J Am Coll Cardiol.

[16] Amat-Santos I.J., Messika-Zeitoun D., Eltchaninoff H. (2015). Infective endocarditis after transcatheter aortic valve implantation: results from a large multicenter registry. Circulation.

[17] Del Val D., Panagides V., Mestres C.A., Miro J.M., Rodes-Cabau J. (2023). Infective endocarditis after transcatheter aortic valve replacement: JACC state-of-the-art review. J Am Coll Cardiol.

[18] Otto C.M., Nishimura R.A., Writing Committee Members (2021). 2020 ACC/AHA guideline for the management of patients with valvular heart disease: a report of the American College of Cardiology/American Heart Association Joint Committee on Clinical Practice Guidelines. J Am Coll Cardiol.

[19] Habib G., Derumeaux G., Avierinos J.F. (1999). Value and limitations of the Duke criteria for the diagnosis of infective endocarditis. J Am Coll Cardiol.

[20] Dickerman S.A., Abrutyn E., Barsic B. (2007). The relationship between the initiation of antimicrobial therapy and the incidence of stroke in infective endocarditis: an analysis from the ICE Prospective Cohort Study (ICE-PCS). Am Heart J.

[21] Pericas J.M., Llopis J., Athan E. (2021). Prospective cohort study of infective endocarditis in people who inject drugs. J *Am Coll Cardiol*.

[22] Cosgrove S.E., Vigliani G.A., Fowler V.G. (2009). Initial low-dose gentamicin for Staphylococcus aureus bacteremia and endocarditis is nephrotoxic. Clin Infect Dis.

[23] Iversen K., Ihlemann N., Gill S.U. (2019). Partial oral versus intravenous antibiotic treatment of endocarditis. N Engl J Med.

[24] Baddour L.M., Wilson W.R., Bayer A.S. (2015). Infective endocarditis in adults: diagnosis, antimicrobial therapy, and management of complications: a scientific statement for healthcare professionals from the American Heart Association. Circulation.

[25] Siddiqui E., Alviar C.L., Ramachandran A. (2022). Outcomes after tricuspid valve operations in patients with drug-use infective endocarditis. Am J Cardiol.

[26] Mack M.J., Lancellotti P. (2020). Early surgery in infective endocarditis: can it be too early?. J Am Coll Cardiol.

[27] Werdan K., Dietz S., Loffler B. (2014). Mechanisms of infective endocarditis: pathogen-host interaction and risk states. Nat Rev Cardiol.

[28] Elgharably H., Hussain S.T., Shrestha N.K., Blackstone E.H., Pettersson G.B. (2016). Current hypotheses in cardiac surgery: biofilm in infective endocarditis. Semin Thorac Cardiovasc Surg.

[29] Scantland J., Hendrix J., Schmitz A., Casciani T., Butty S. (2023). Clinical efficacy of percutaneous vegetectomy in tricuspid and right-heart indwelling device infective endocarditis. Angiology.

[30] Veve M.P., Akhtar Y., McKeown P.P., Morelli M.K., Shorman M.A. (2021). Percutaneous mechanical aspiration vs valve surgery for tricuspid valve endocarditis in people who inject drugs. Ann Thorac Surg.

[31] Zhang R.S., Alam U., Maqsood M.H. (2023). Outcomes with percutaneous debulking of tricuspid valve endocarditis. Circ Cardiovasc Interv.

[32] Akhtar Y.N., Walker W.A., Shakur U., Smith G., Husnain S.S., Adigun S.F. (2021). Clinical outcomes of percutaneous debulking of tricuspid valve endocarditis in intravenous drug users. Catheter Cardiovasc Interv.

[33] Starck C.T., Schaerf R.H.M., Breitenstein A. (2020). Transcatheter aspiration of large pacemaker and implantable cardioverter-defibrillator lead vegetations facilitating safe transvenous lead extraction. Europace.

[34] Durante-Mangoni E., Ursi M.P., Andini R. (2022). Long-term outcome of infective endocarditis involving cardiac implantable electronic devices: impact of comorbidities and lead extraction. J Clin Med.

[35] Sanfilippo A.J., Picard M.H., Newell J.B. (1991). Echocardiographic assessment of patients with infectious endocarditis: prediction of risk for complications. J Am Coll Cardiol.

[36] Thuny F., Di Salvo G., Belliard O. (2005). Risk of embolism and death in infective endocarditis: prognostic value of echocardiography: a prospective multicenter study. Circulation.

[37] Akhtar Y.N., Barry IvN., Foster M.T. (2022). Case series of percutaneous mechanical aspiration of mitral valve endocarditis. J Am Coll Cardiool Case Rep.

[38] Chu V.H., Park L.P., Athan E. (2015). Response to letter regarding article, “Association between surgical indications, operative risk, and clinical outcome in infective endocarditis: a prospective study from the International Collaboration on Endocarditis”. Circulation.

[39] Tarakji K.G., Wazni O.M., Harb S., Hsu A., Saliba W., Wilkoff B.L. (2014). Risk factors for 1-year mortality among patients with cardiac implantable electronic device infection undergoing transvenous lead extraction: the impact of the infection type and the presence of vegetation on survival. Europace.

[40] Renew J.R., Wittwer E.D., Robb T.M., Fritock M.D. (2016). AngioVac removal of a saddle pulmonary embolus using TEE guidance and venoarterial ECMO support. J Cardiothorac Vasc Anesth.

[41] Kang D.H., Kim Y.J., Kim S.H. (2012). Early surgery versus conventional treatment for infective endocarditis. N Engl J Med.

[42] Fosbol E.L., Park L.P., Chu V.H. (2019). The association between vegetation size and surgical treatment on 6-month mortality in left-sided infective endocarditis. Eur Heart J.

